# Estimation of optimal number of gates in dual gated ^18^F-FDG cardiac PET

**DOI:** 10.1038/s41598-020-75613-5

**Published:** 2020-11-09

**Authors:** R. Klén, J. Teuho, T. Noponen, K. Thielemans, E. Hoppela, E. Lehtonen, H. T. Sipila, M. Teräs, J. Knuuti

**Affiliations:** 1 Turku PET Centre, Turku University Hospital, University of Turku, Turku, Finland; 2 Division of Medical Imaging, Department of Medical Physics, Turku University Hospital, Turku, Finland; 3 Department of Clinical Physiology and Nuclear Medicine, Turku University Hospital, Turku, Finland; 4 University College London, London, UK; 5 Hammersmith Imanet Ltd, London, UK; 6 Department of Future Technologies, University of Turku, Turku, Finland; 7 Institute of Biomedicine, University of Turku, Turku, Finland

**Keywords:** Imaging techniques, Cardiovascular diseases

## Abstract

Gating of positron emission tomography images has been shown to reduce the motion effects, especially when imaging small targets, such as coronary plaques. However, the selection of optimal number of gates for gating remains a challenge. Selecting too high number of gates results in a loss of signal-to-noise ratio, while too low number of gates does remove only part of the motion. Here, we introduce a respiratory-cardiac motion model to determine the optimal number of respiratory and cardiac gates. We evaluate the model using a realistic heart phantom and data from 12 cardiac patients (47–77 years, 64.5 on average). To demonstrate the benefits of our model, we compared it with an existing respiratory model. Based on our study, the optimal number of gates was determined to be five respiratory and four cardiac gates in the phantom and patient studies. In the phantom study, the diameter of the most active hot spot was reduced by 24% in the dual gated images compared to non-gated images. In the patient study, the thickness of myocardium wall was reduced on average by 21%. In conclusion, the motion model can be used for estimating the optimal number of respiratory and cardiac gates for dual gating.

## Introduction

Image quality in cardiac positron emission tomography (PET) is reduced by respiratory and cardiac motions. The resolution and contrast of PET images are reduced when the motion amplitude is above the full-width half maximum (FWHM) of the PET scanner^1^. Thus, motion introduces image blurring, resulting to loss of small details and image contrast. In most ^18^F-FDG patient studies, the respiratory and cardiac motion amplitudes have been determined to be more than 5 mm, which is above the FWHM of modern PET scanners^1^. The benefit of motion compensation is especially important for imaging of the coronary plaques, as recent studies have shown for ^18^F-NaF^2,3^. Thus, methods to estimate the amount of motion and minimise its effects to detection of small targets, such as coronary plaques are needed.

To reduce motion effects, numerous gating methods have been introduced^4–6^, where PET raw data is divided into smaller time frames according to a motion. The motion signal can be obtained by tracking the respiration or the heartbeat. For respiratory motion tracking, various methods have been introduced^7–9^. A recent summary of the methodologies available can be found from the following reviews by^10,11^. For cardiac motion tracking, ECG still remains the most common option.

In clinical practice, only respiratory or cardiac gating is usually applied instead of using both simultaneously. Cardiac gating is routinely used for cardiac 18F-FDG imaging. In a single gating study, a respiratory gate will contain residual cardiac motion while a cardiac gate will contain residual respiratory motion. Ideally, the effect of both motions can be minimised. For this purpose, dual gating has been introduced^5,12–19^, where simultaneous respiratory and cardiac gating is applied. Motion correction methods have also been extensively studied^20–24^, however they are not yet widely applied in clinical practice.

In principle, increasing the number of gates enables to capture motion in each gate with increased accuracy, as each gate contains only a small fraction of the total motion seen in non-gated images. However, while this reduces motion effects as a function of the number of gates, the signal-to-noise ratio (SNR) will be reduced as well which might cause variation in quantitative measurements^25^ and result to a loss of contrast in small targets, such as coronary plaques. To compensate for the reduced SNR, the acquisition time needs to be extended or the number of gates needs to be reduced.

Thus, a balance between optimal image quality and captured motion must be found to determine the optimal number of gates. Moreover, to determine the amount of motion from the gated images, the motion in the gated images needs to be modelled in terms of respiration, cardiac contraction and their combination^26^. The optimal number of gates has been investigated previously in the literature for respiratory gating in oncology^27^ and cardiology^26^. However, authors are not aware of any previous studies to determine the optimal number of dual gates, for both cardiac and respiratory gates with motion modelling. Moreover, the number of gates used in dual gated PET studies is much varied. The number of gates used were four respiratory and eight cardiac gates in^28^, six respiratory and three cardiac gates in^29^, three respiratory and four cardiac gates in^15,19^, eight respiratory and four and six cardiac gates^16^, four respiratory and four cardiac gates^3^ and eight respiratory and eight cardiac gates in^13^.

Therefore, there is a dire need to develop modelling approaches which can be applied to define the optimal number of dual gates in patient studies. In this study, we developed and assessed such an approach by motion measurement and modelling to estimate the optimal number of dual gates in a phantom and a patient study. For measurement of respiratory motion, we propose to use the spirometric volume, which has been shown to change linearly in terms of the motion of the myocardium in cardiac PET^30^. Furthermore, spirometric measurements have been proposed to have potential to guide selection of optimal number of respiratory gates in cardiac PET^30^. In this study, motion from respiration, cardiac contraction and their combination was modelled and validated with a moving heart phantom to derive the optimal number of respiratory and pulsatile gates. The motion models were applied to an ^18^F-FDG coronary plaque study to determine the optimal number of dual gates in a group of patients. To the best of our knowledge, this is the first study to implement a dual respiratory-cardiac motion model in cardiac PET.

## Materials and methods

### Phantom and patient studies

Phantom and patient studies were performed on a GE Discovery VCT PET/CT (GE Healthcare, Milwaukee, US). The study protocol was approved by the Ethics Committee of the Hospital District of Southwest Finland. All methods were carried out in accordance with relevant guidelines and regulations. The detailed performance evaluation of the scanner can be found in^31^. The scanner was equipped with a spirometry device (S/5 anaesthesia monitor, GE Healthcare) and the Varian Respiratory Position Monitor (RPM) (Varian Medical Systems, Palo Alto, CA, USA) for respiratory gating. The patients used an airtight breathing mask that was connected to S/5 anaesthesia monitor to which an airflow signal was stored. An IVY Biomedical 3150-B ECG monitor was used for cardiac gating. The scanner has an effective resolution of 5.2 mm and axial field of view (FOV) of 15.7 cm. More technical details of the scanner can be found in^31^. The technical details including the set-up and validation of the spirometry device for respiratory gating can be found in the article by Kokki et al.^30^.

In the phantom study, a realistic heart phantom with respiratory and cardiac motion was used^19^. The phantom^32^ contains an outer and inner plastic balloon, which are nested and filled with radioactive water. The inner balloon inflates and deflates to simulate pulsatile motion. The diameter of the outer balloon changes 7 mm at maximum. The balloons are also moved mechanically in axial direction, simulating respiration. The resulting respiratory motion is 20 mm. Radioactive point sources were attached to the outer balloon to simulate coronary plaques of 3 mm in size. The phantom is programmed to send both respiratory and cardiac triggers at intervals corresponding to respiration and pulsatile motion to the PET/CT system. Detailed description of the phantom study with figures is given in^19^.

In the patient study, the data of 12 cardiac patients was used. The full details of the patient cohort can be found in Table 1. In short, the patient cohort consisted of 12 patients, eight males and four females, aged between 47–77 years and 64.5 years on average. An average dose of 350 MBq was prescribed and the imaging was started approximately 125 min after injection. The subjects were referred to a dual gated PET/CT scan as a part of a larger clinical study and gave their informed consent. All subjects were suspected of vulnerable coronary plaques and were prescribed a diet to suppress the uptake of ^18^F-FDG in the myocardium^33^. The patients where the dieting was not successful (N = 5) to supress the myocardium uptake are also marked in Table 1.

**Table 1:** Characteristics of the patient cohort included in the study.

Patient number	Sex (M/F)	Age (Y)	Weight (kg)	Height (m)	Dose (MBq)	Prompts (count)	Myocardial uptake (X = Yes)
1	M	70	80	1.74	370	4.24E + 08	X
2	M	64	96	1.67	398	2.82E + 08	–
3	F	57	60	1.61	356	6.57E + 08	X
4	M	70	84	1.74	341	4.34E + 08	X
5	M	56	90	1.83	341	2.43E + 08	–
6	F	77	58	1.59	352	5.66E + 08	X
7	M	71	80	1.70	394	2.21E + 08	X
8	F	57	70	1.67	355	4.71E + 08	–
9	M	47	120	1.80	362	2.70E + 08	–
10	F	76	72	1.61	340	4.49E + 08	X
11	M	62	106	1.79	355	3.94E + 08	X
12	M	67	101	1.81	232	2.00E + 08	–

During the PET/CT imaging, two of the scans had technical problems and could not be used in the analysis of image quality, specifically the measurement of SNR. Patient 2 experienced a mismatch between PET and CTAC scans resulting to erroneous attenuation correction. Patient 7 had to be excluded due to overestimation in scatter correction, resulting to large photophenic regions in the lung.

The imaging protocol was identical for both phantom and patient studies. In the patient study, average dose was less than 12 mSv, with 4 mSv due to 4D CT. PET/CT protocol consisted of a CT-based attenuation correction (CTAC) with 120 kV and max 80 mA, followed by gated ^18^F-FDG PET of one bed position covering the thorax, followed by 4D CT. PET acquisitions were collected in list-mode with acquisition time of 30 min. Respiratory signals were recorded simultaneously with the RPM and the S/5 anaesthesia monitor at sampling frequency of 25 Hz, while ECG signal was recorded with the ECG monitor. Both the RPM and ECG monitors were set to insert respiratory and cardiac triggers to the list-mode data. Before gating, PET list-mode data and the volume signal from the spirometry device were synchronized using trigger time information in RPM and list mode data files and cross correlation spectrum between RPM and volume signals. The synchronisation quality of the signals was confirmed visually. The spirometry signal was qualitatively evaluated on basis of signal quality, breathing irregularities and signal artefacts by an experienced nuclear medicine physicist with nearly 10 years of experience in gated PET studies. As the spirometry is a zero-baseline signal, patient motion was evaluated based only on the RPM signal. In addition, the amount of rejected data based on the spirometry signal was calculated. The signal evaluation is found in Table 2.

**Table 2:** Evaluation of the respiratory signal quality using spirometry with the amount of rejected data based on the spirometry signal.

Patient number	Signal quality (1—5)	Rejected data (%)	Breathing irregularity	Signal artefacts	Patient motion (RPM)
1	5	15.86	Very minor	–	–
2	4	10.64	Minor	Motion	Yes
3	5	20.67	Minor	–	–
4	5	12.92	Very minor	–	–
5	5	5.99	Minor	–	–
6	5	14.55	–	–	Yes
7	2	42.90	Long cycle length	Lost signal	Yes
8	1	23.73	–	Lost signal	–
9	5	14.36	Minor	–	–
10	2	31.13	Moderate	Clipping	–
11	3	21.39	Minor	Clipping	–
12	4	27.78	Minor	–	–

The patient gross motion during the study was valuated based on the RPM signal.

Gating the data into dual gates was performed with an offline computer using a software package provided by GE Healthcare (RGT, Research Gating Tool). The gating method is described in detail in the paper of Kokki et al.^19^ whereas the data processing for the spirometry data is described in^30^. Only the data considered as valid by the spirometry system was used in gating and motion modelling.

### Selection of dual gating methods

Amplitude-based respiratory gating and phase-based cardiac gating were used for dual gating. Amplitude-based gating is expected to perform better if the respiratory signal includes cycle variations^26^. Phase-based gating for cardiac gating is considered to be a clinical standard^34^.

The respiratory signal was divided to gates of equal height according to measured lung volume amplitude. The respiratory gating method is described in more detail in^30^. In cardiac gating, the ECG cycle was divided into gates which had equal length according to time. The cardiac gate lengths were determined according to the length of the cardiac cycle and the number of cardiac gates used. After combining respiratory and cardiac gates to dual gates, an equal relative displacement according to lung volume signal and equal time according to R-peak interval is contained in each dual gate.

To determine the optimal number of gates, the phantom study was used with a wide coverage of gates, from no motion capture (single gate) to maximal motion capture (60 gates). The number of respiratory gates used was 1, 2, 3, 4, 5 and 6 while the number of cardiac gates was 1, 4, 6, 8 and 10. The number of gates for the patient study was then determined based on the phantom study, resulting to 1, 3, 4 and 5 respiratory gates and 1, 6 and 8 cardiac gates.

### Image reconstruction

Reconstruction software provided by GE Healthcare was used for reconstruction. An iterative three-dimensional ordered subsets expectation maximisation algorithm was used with two iterations, 28 subsets and 6 mm Gaussian post-filtering. The size of the reconstructed images was $192\times 192\times 47$ with a voxel size of $1.82 \mathrm{mm}\times 1.82\mathrm{ mm}\times 3.27\mathrm{ mm}$ and a 35 cm transaxial FOV. The reconstruction parameters are similar to those in clinical studies at our institution.

Attenuation correction was performed with 4D CT images, where the corresponding amplitude of respiration in PET and CT was used for attenuation correction^35,36^. All necessary quantitative corrections for decay, dead-time, scatter, randoms, detector geometry and normalisation were performed.

### Modelling and analysis workflow

**Figure 1:**
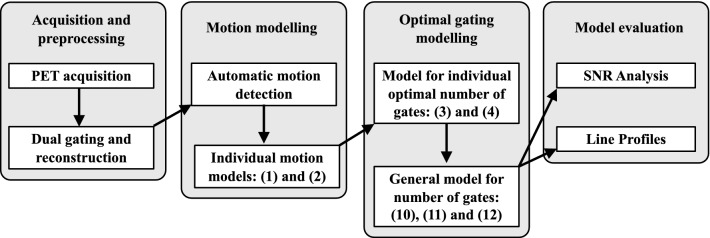
Modelling and analysis workflow. The motion modelling consists of the following 4 steps. Step 1: Acquisition and dual gating of the PET scans with up to 60 gates. Step 2: Automatic motion detection and individual motion models for respiratory, cardiac and dual motion. Step 3: Individual and general models for number of gates in terms of respiratory, cardiac and dual motion. Step 4: Evaluation of the general model for optimal number of gates using SNR and line profiles. General models were built only for the patient study. Numbers in parenthesis refer to the models defined by the corresponding equations.

The general workflow for determining the optimal number of gates is depicted in Fig. 1. The modelling and analysis start with dual gating of PET images with various number of gates for the phantom and each subject. Then, from each dual gated image, respiratory and pulsatile motions are extracted using automated segmentation. Based on extracted motion, models for motion as function of number of gates are created for each subject. The models are used to estimate total extent of motion and optimal number of gates for each subject. Finally, the optimal number of gates and total motion for each subject are used to define a general model for optimal number of gates in terms of motion. In the following sections, we describe the workflow in more detail.

### Measurement of respiratory and cardiac motion

To derive a motion estimate to be used for model fitting in Section “Models for motion estimation”, respiratory and cardiac motions were first measured between gated images. For the phantom, the true reference motion was known^32^ and was used to validate the accuracy of the motion model and the modelled motion estimate. True respiratory motion was 20 mm and cardiac motion 7 mm.

Respiratory gates corresponding to peak-inspiration and end-expiration were used for to measure the motion between different respiratory states. Similarly, cardiac motion was measured by comparison between systolic, mid-diastolic and diastolic gates, where the start and end of the cardiac cycle was compared to mid-cycle.

In the phantom study, the maximal respiratory motion was determined by measuring the location of the hot spots between gated images. A local maximum and a weighted average of local maximum with threshold of 90% were used to locate the centre of hot spots in each gated image. Thereafter, the motion could be measured in mm by determining the difference of the centres of the hot spots between subsequent motion states.

In patient studies, a similar approach was applied. The respiratory and cardiac motion was determined from the motion of the centre of mass of the myocardium (CMA). The method involves automatic extraction of the myocardium activity and the calculation of the centre of mass, as described originally by^22^ and used by ^30,37^. The difference of CMA between different cardiac and respiratory states was then used to determine the motion in mm between motion states.

Due to the low quality of the dual gated PET images, respiratory- and cardiac-averaged images were used for motion determination. Respiratory-averaged images were created by averaging all gated images in a gating scheme over all respiratory phases. In a similar manner, cardiac-averaged images were created by averaging all gated images in a gating scheme over all cardiac phases. Thereafter, respiratory motion was defined by comparing the motion of CMA in cardiac-averaged images. Cardiac motion was determined by comparing the motion of CMA in respiratory-averaged images. While this approach introduces a small amount of blurring to the images, it allows to reduce the effect of residual noise to the CMA. As a quality control, the location of CMA was confirmed by investigating a fusion image of the location of the calculated CMA and the extracted myocardium.

### Models for motion estimation

The final motion amplitude was estimated by motion modelling and fitting to motion data measured in Section “Measurement of respiratory and cardiac motion”. As described previously, when a sufficient number of gates are used for motion capture, an empirical motion model can be derived and fitted to the measured motion data from those gates^26^. The derived model can then be used to deliver a general motion estimate for determining the optimal number of gates as a function of motion amplitude. We also improve on the model of^26^ which allows to estimate respiratory motion only, while the models presented in this paper can be used for respiratory, cardiac and dual gating. For detailed comparison with the model in paper of Dawood et al*.*^26^ and the analytical model presented in this paper, please see section “Comparison with an existing motion model”.

Here, we propose a simple analytical model which can be used for both respiratory and cardiac motion modelling. The model is based on the assumption that both cardiac and respiratory motions are cyclic motions with average amplitude of *h*. Thus, if the data is gated into *n* gates, then two sequential gates can be assumed to detect motion of approximately $h/n$. Since *n* gates form *n-*1 intervals between midpoints of the gates, the total motion detected by *n* gates is $h(n-1)/n.$ Therefore, the assumption is that motion is divided into equal patterns or intervals defined by the total gate number *n.* In practice, this would require that each respiratory or cardiac gate would be divided in such a way that each gate would contain an equal amount of motion for the assumption to be valid.

However, in practice, the motion cannot be divided into perfect intervals which contain equal amount of motion. This is due to changes in respiratory and cardiac patterns during the study and inherent inaccuracies in motion signal measurements and gating. In practice, only the total motion $f\left(r\right)$ or $f\left(p\right)$ and the total number of gates $\left(r,p\right)$ which are used to capture that amount of motion can be assumed to be known accurately. The total motions $f\left(r\right)$ and $f\left(p\right)$ can then be measured between the two extreme gates which contain the maximal change in either the respiratory or cardiac pattern (e.g. between the peak inhalation and end-expiratory states) using any arbitrary gating pattern. The accuracy of the total motion estimate is then dependent only on the total number of gates used to capture the maximal amount of motion between the first and the last gate and not on exact gate division.

The proposed model is thus based on the assumption that using a number of respiratory or cardiac gates $\left(r,p\right)$ a total amplitude of motion $f\left(r\right)$ or $f\left(p\right)$ can be measured accurately and the effects of respiratory and cardiac motions can be summed together. This approach follows intuitively the motion effects seen in PET images, where the combined effects of respiratory- and pulsation-introduced image blurring are non-differentiable. In this case, a general model for either respiratory or cardiac gating can be described as:

1 $$ f\left( r \right) = \frac{{c*\left( {r - 1} \right)}}{r},f\left( p \right) = \frac{{c*\left( {p - 1} \right)}}{p}, $$

where functions $f\left(r\right)$ and $f\left(p\right)$ give the total amplitude of respiratory and pulsatile motion as measured in the gated images, *r* is the number of respiratory, and *p* is the number of cardiac gates and parameter *c* is obtained from the fitting, describing the motion amplitude for either respiratory and cardiac motion.

Since respiratory and cardiac motions occur independently, a model for dual gating can be obtained from (1) modelling both the respiratory (*r*) and pulsatile (*p*) components separately with individual number of gates defined as *r* and *p*. The total motion *c* = *a* + *b* can be described as individual components of respiratory motion *a* and cardiac motion *b*.

As in Eq. (1), we assume that contribution of respiratory and cardiac motions is summed together in combined respiratory and cardiac gates, allowing to define the total number of gates as $r*p$ in Eq. (2). This way, the dual motion model can be described as:

2 $$g\left(r,p\right)=\frac{a*\left(r-1\right)*p+b*r*(p-1)}{r*p},$$

where function $g(r,p)$ gives the total contribution of respiratory and cardiac motion (total motion) in mm as measured in the gated images, *r* is the number of respiratory gates, *p* is the number of cardiac gates and *a* and *b* are the motion estimates obtained from the fitting, which can be used to derive the motion amplitude. The parameters *a*, *b* and *c* in (1) and (2) were attained by using the curve fitting toolbox in MATLAB 7.7.0 (MathWorks Inc., Natick, MA, US).

### Models for estimation of optimal number of respiratory and cardiac gates

Increasing the number of gates allows to reduce the motion more effectively in each gate and to increase the motion amplitude measurable from gated images^22^. However, increasing the number of gates decreases the number of events per gate leading to increase of noise. Thus, the optimal number of gates is determined by motion minimisation and increase of noise. We first determine the optimal number of gates for motion minimisation and later we consider the optimal number of gates in terms of noise increase.

The model for optimal number of gates in terms of total motion amplitude for respiratory and cardiac gating can be derived from Eqs. (1) and (2) by using an estimate of image motion and the resolution of the PET scanner as criteria for optimising the model output. Model (1) can be applied for respiratory or cardiac gating only while model (2) can be applied for dual gating.

The total estimated motion of model (1) can be obtained by letting the number of gates to increase limitless. If $f(r)$ gives the amplitude of motion in terms of number of gates *r*, then the total amplitude of motion is determined by ${m}_{est}=\underset{r\to \infty }{\mathrm{lim}}f(r)$. The amplitude of total motion by model (1) is ${m}_{est}=c$. By model (2) the amplitude of total motion is ${m}_{est}=\underset{r\to \infty ,p\to \infty }{\mathrm{lim}}g\left(r,p\right)=a+b$.

The total estimated motion ${m}_{est}$ is an asymptotic value obtained from the model (1) or (2). Thus, we consider the optimal number of gates, as in^26^, to be such that the total motion ${m}_{est}$ is captured up to one-half of the scanner resolution $s$. In other words, we need to find the number of gates such that captured motion is ${m}_{est}-s/2$ or more. For more details, please see Supplementary File. For model (1) the optimal number of gates ${r}_{opt}$ can be formulated as:

3 $${r}_{opt}=\lceil{f}^{-1}({m}_{est}-s/2)\rceil, $$

where $\lceil \, \rceil$ denotes the ceiling function, ${m}_{est}$ is the motion given by model (1) and ${f}^{-1}$ is the inverse function of $f$ described in Eq. (1). For (1), $f$ gives the total respiratory or cardiac motion in mm.

For dual gating, model (2) needs to be applied to include both cardiac and respiratory motion for estimation of the optimal number of gates. Thus, in dual gating we obtain:

4 $$\left({r}_{opt},{p}_{opt}\right)=\lceil{g}^{-1}({m}_{r}-s/2,{m}_{p}-s/2)\rceil,$$

where $g^{ - 1}$ is the inverse function of (2), $m_{r} = a$ and $m_{p} = b$ are the total respiratory and pulsatile motion obtained from (2), and *s* is the scanner resolution.

Equation (4) has multiple solutions due to different combinations of cardiac and respiratory gates. Thus, a solution is desired where the total number of gates is minimal to compensate a specific motion amplitude. The optimal number can be derived by finding a number of respiratory gates ${r}_{opt}$ and cardiac gates ${p}_{opt}$ such that product ${r}_{opt}*{p}_{opt}$ is minimal and the total number of gates is sufficient to minimise a total motion of *m*_*est*_. The method to determine the optimal number of gates for the phantom and patient study is described in detail in Section “Determining the optimal number of gates in phantom and patient study”.

### Validation of motion estimation models

The motion was measured from dual gated PET images and was compared to the motion estimated by the models (1) and (2). The motion estimation accuracy of the models was first validated by using phantom data, which served as a reference standard for model performance. Thereafter, the models were applied to the patient data by using the measured patient motion as input.

To compare the results, mean difference (MD):

5 $$MD=\frac{1}{n}\sum \left|{x}_{i}-{y}_{i}\right|,$$

and root-mean-square error (RMSE) were used:

6 $$RMSE=\sqrt{\frac{1}{n}\sum {({x}_{i}-{y}_{i})}^{2}},$$

where $x=\left({x}_{1},{x}_{2},\dots ,{x}_{n}\right)$ and $y=\left({y}_{1},{y}_{2},\dots ,{y}_{n}\right)$ are data sets corresponding to measured motion from the images and estimated motion from the model, respectively.

We report MD and RMSE to evaluate the difference between measured motion and motion estimated by the models (1) and (2) in phantom and patient studies.

### Determining the optimal number of gates in phantom and patient study

After the validation of the motion estimation accuracy with the phantom, we used the motion estimates from model (1) and (2) as input to (3) and (4) determine the optimum number of gates needed to minimise the motion in the phantom and in each individual subject. Thereafter, a generalised model for the optimum number of gates was derived.

For the phantom study, the optimum number of gates was determined as combination of gates, which produces the highest motion minimisation with the minimum number of gates as ${r}_{opt}*{p}_{opt}$.The motion minimisation threshold was calculated as *m*_*est*_ = 12.7 mm by using a respiratory motion of 14.1 mm and a cardiac motion of 3.8 mm (total motion of 17.9 mm) and scanner resolution of 5.2 mm, as 14.1 mm − 2.6 mm + 3.8 mm − 2.6 mm = 12.7 mm. For the phantom study, we report the total motion amplitude as function respiratory and cardiac gates from model (2) and the optimal number of gates given by model (4).

For the patient study, the motion derived from models (1) and (2) was used as input for (3) and (4), and the optimal number of gates for each patient was determined. For determining the respiratory and pulsatile motion separately, model (1) was used. For dual gating, model (2) was used to estimate the total motion. It should be noted that model (3) estimates the number of respiratory and cardiac gates separately for each motion while model (4) gives the total number of dual gates directly based on total motion, which can be used to determine the optimal number of respiratory and cardiac gates.

Thereafter, the estimated motion from models (1) and (2) were plotted against the optimal number of gates estimated with (3) and (4). For model (1), respiratory and cardiac motions were plotted against the optimum number of respiratory and cardiac gates from (3). For model (2), the total motion given by the model was plotted against the number of dual gates estimated with (4). Finally, a generalised model for the optimal number of gates for respiratory, cardiac and dual gating was derived by fitting to the data by linear regression.

For the patient study, we report the optimal number of respiratory and cardiac gates given by model (3) and the total number of gates given by model (4) and the resulting optimal combination of respiratory and cardiac gates specific for motion amplitude of each patient.

In addition, we report the general models for respiratory, cardiac and dual gating as derived by fitting of the data. Finally, we report the goodness-of-fit for the general models for cardiac, respiratory and dual gating to estimate the number of gates.

### Signal to noise ratio evaluation for optimal number of gates

In addition to motion minimisation, the second limiting condition for optimal number of gates is the image quality. For an image quality metric, the global SNR inside a gating scheme was used as a function of the total number of dual gates derived from (4). SNR is used to monitor the image quality as the number of gates is increased and to evaluate that the value does not decrease on average more than 3 dB from non-gated image to the optimally gated images.

The SNR measurements were performed using the method applied in previous studies^26^. In short, the SNR is defined from a VOI in a background region where the PET signal is assumed to be quite homogeneous. In the patient study, the lung region was used as the background region whereas the inner balloon was used in the phantom study. An ellipsoid-shaped VOI was placed inside the inner balloon in the phantom study. In the patient study, a cuboid VOI was placed inside the right lung similarly to^38^. Sizes of the VOIs were: $13\times 13\times 13$ pixels in the phantom study and $10\times 15\times 15$ pixels in the patient study.

The SNR was defined as the mean of voxels divided by standard deviation of the voxels in a homogeneous volume of interest (VOI):

7 $$SNR=10*{\mathrm{log}}_{10}\frac{\overline{x}}{\sqrt{{(\sum ({x}_{i}-\overline{x}))}^{2}}/N},$$

where $\overline{x}$ is mean value of voxels ${x}_{i}$ in the background VOI and $N$ is the number of voxels in the VOI. For each gated image set, the mean SNR is reported for the summed image. By summation of the images of the gated image set, the noise is also added to the summed image as the noise in the individual gates is increased. Thus, this metric allows to determine the SNR with a high number of gates and corresponds to the average SNR inside each gating scheme, although the SNR in individual gates is expected to be lower than this value.

To verify the dependency of SNR on the number of gates, a SNR curve $a+b*{\mathrm{log}}_{10}(n)$ as in^26^ was fitted to the SNR data, where $n$ is the number of gates. We report SNR versus the total number of gates in phantom and patient studies. As specified in Section “Phantom and patient studies”, patients 2 and 7 had to be omitted from the SNR analysis due to technical reasons.

### Evaluation of image quality

To evaluate the image quality with optimal number of gates, manually selected profiles for non-gated and gated images were extracted from a single plane of a single gate. In the phantom study, the profile was drawn over the most active hot spot. In the patient study, the profile was drawn so that it overlaps with both the septal and lateral wall of the myocardium. The gates selected for both the phantom and patient analysis were the last gates of each individual gating schema e.g. gate 20 of the 5,4 *(r,p)* gating scheme, containing the most optimal gate in terms of counting statistics saved with the best motion minimisation qualities.

The peak of the profiles was used to calculate the full width at half maximum (FWHM). The relative difference of FWHM between gated and non-gated images was used as measurement for image quality and improvement in motion minimisation. The relative difference was calculated as follows:

8 $$\mathrm{\%}\Delta ={(FWHM}_{DG}-{FWHM}_{NG}) / {FWHM}_{NG}*100\mathrm{ \%},$$

where ${FWHM}_{DG}$ and ${FWHM}_{NG}$ refer to FWHM measured from dual gated and non-gated images, respectively. Finally, we report the relative difference of FWHM measured from the septal and the lateral myocardial wall.

### Comparison with an existing motion model

Finally, the performance of the proposed models was compared to a previously introduced empirical respiratory motion model^26^. The model can be used for respiratory motion estimation for determining the optimal number of gates. As this model is only appropriate for estimation of respiratory motion, no comparison with the cardiac model or dual cardiac-respiratory motion model could be made. The analytical derivation of the models is given in Supplementary Data.

Dawood et al*.*^26^ proposed the following empirical model for estimating the motion amplitude of the heart due to respiratory motion only:

9 $${f}_{1}\left(r\right)=a+b*{e}^{c/r},$$

where function ${f}_{1}\left(r\right)$ gives the total amplitude of respiratory motion, *r* is the number of respiratory gates and parameters *a*, *b* and *c* are obtained from the fitting. The elementary problem with the model in (9) is the behaviour when *r* is close to 1. When $r=1$ there should be only one gate and thus motion should be 0. However, in (9) the motion is ${f}_{1}\left(1\right)=a+b*{e}^{c}$. The proposed model in (1) fulfils the condition $f\left(1\right)=0.$

For the comparison, we report the model fitting results from the estimated motion in (9) and compare it to the estimated motion by (1) in the phantom study. We also report the number of gates as given by (9) and compare the generalised model from (9) to the respiratory motion model in (10).

## Results

### Validation of motion estimation models by phantom study

The results from the motion estimation by models (1) and (2) are represented in Table 3. All of the motion measurements from phantom PET images using CMA with all gating schemes are reported in Supplementary Data 1. In summary, model (1) had RMSE of 0.44 mm and MD of 0.35 mm for respiratory motion estimation. Model (1) gave an RMSE of 0.21 mm and MD of 0.16 mm when applied for cardiac motion estimation.

**Table 3:** Error in mm of respiratory motion given by models (1) and (2) compared to the reference motion from the phantom, as given in Supplementary Data 1.

Model	Motion	Target	RMSE	MD	Absolute value
(1)	Respiratory	Largest hot spot	0.38	0.30	0.00–0.73
(1)	Respiratory	Average of all	0.44	0.35	0.00–0.78
(1)	Cardiac	Largest hot spot	0.09	0.07	0.00–0.12
(1)	Cardiac	Average of all	0.21	0.16	0.00–0.33
(2)	Total	Largest hot spot	0.83	1.43	0.00–0.98
(2)	Total	Average of all	0.93	1.70	0.00–0.90

The RMSE and MD are given as an error over all gating schemes. Absolute value refers to the absolute difference of estimated motion from the model and the reference motion given as a range over all gating schemes. The number of respiratory gates used was 1, 2, 3, 4, 5 and 6 while the number of cardiac gates was 1, 4, 6, 8 and 10.

In estimation of the total motion from respiration and pulsation for dual gating, model (2) gave an RMSE of 0.93 mm and MD of 1.70 mm. In all cases estimation of the largest hot spot gave more accurate results than estimating all hot spots on average (Supplementary Data 1).

### Determining the optimal number of gates as function of motion—phantom study

The total motion amplitude as function respiratory and cardiac gates from models (2) and (4) is represented in Table 4. The combinations of cardiac and respiratory gates for efficient motion capture in terms of scanner resolution are highlighted in red. Therefore, based on (4) and Table 4 the optimal number is 5 respiratory and 4 cardiac gates, which is needed to minimise motion up to 12.7 mm in amplitude.

**Table 4:** Estimated motion in mm as a function of respiratory and pulsatile gates in the phantom study.

Total motion in mm		Number of respiratory gates					
		1	2	3	4	5	6
Number of pulsatile gates	1	0	7.5	10.4	10.6	11.1	12.0
	4	3.0	9.3	11.2	12.5	***12.7***	*12.8*
	6	3.1	9.3	11.3	12.5	*12.8*	*12.9*
	8	3.5	9.4	11.8	12.6	*12.8*	*12.9*
	10	3.5	9.4	11.8	12.6	*12.8*	*12.9*

Numbers in italic font signify the number of gates needed to capture maximum motion amplitude of 12.7 mm. Bold font indicates the optimal number of gates, where the product of respiratory and cardiac gates is minimal.

### Applying the motion estimation models to a patient study

The results of motion estimation with different models compared to motion measured from PET images in the patient study are presented in Table 5. All measured motions from gated PET images using CMA in the patient study with the estimated respiratory and cardiac motion amplitudes from models (1) and (2) are shown in Supplementary Data 2.

**Table 5:** Error in mm of the motion estimated by model (1) and (2) compared to the measured motion from the PET images.

Patient number	RMSE			MD		
	(1)	(1)	(2)	(1)	(1)	(2)
	Resp.	Card	Dual	Resp.	Card	Dual
1	0.09	0.12	0.23	0.07	0.09	0.20
2	0.30	0.23	1.18	0.24	0.19	0.82
3	0.03	0.04	0.11	0.02	0.04	0.10
4	0.16	0.07	0.33	0.14	0.04	0.28
5	0.33	0.06	0.45	0.24	0.05	0.35
6	0.14	0.15	0.21	0.11	0.12	0.18
7	0.06	0.15	0.18	0.05	0.1	0.13
8	0.86	1.09	2.53	0.71	0.73	1.76
9	0.32	0.12	1.87	0.25	0.08	1.44
10	0.25	0.39	3.65	0.43	0.32	3.30
11	0.07	0.03	0.16	0.07	0.02	0.14
12	0.25	0.71	0.68	0.25	0.56	0.57

Left column shows the patient number.

For the patient data, respiratory motion varied between 2.5 mm and 17.8 mm. Cardiac motion varied between 5.5 mm and 13.0 mm (Supplementary Data 2). Respiratory motions and cardiac motions were 12.0 mm and 9.9 mm on average. For nearly all the patients, estimated dual and respiratory motions were larger than the measured motion. For patients 2 and 9 the estimated motion was smaller than the measured motion (Supplementary Data 2).

The RMSE between the measured and the estimated respiratory motion on average was 0.27 mm for model (1). The average MD between the measured and the estimated respiratory motion 0.20 mm for model (1). For cardiac motion estimation, model (1) gave an RMSE of 0.20 mm and MD of 0.16 mm.

Model (2) showed similar behaviour in the patient study as in the phantom study. The average RMSE between the measured and the estimated total motion for model (2) was 0.97 mm, while the average MD was 0.77 mm.

### Determining the optimal number of gates as function of motion—patient study

The results for the optimal number of dual gates estimated based on models (1) and (2) and models (3) and (4) are presented in Table 6.

**Table 6:** Optimal number of gates for each patient by models (3) and (4).

Patient number	1	2	3	4	5	6	7	8	9	10	11	12
Model (3)												
Respiratory	5	5	4	4	6	5	2	6	5	5	6	9
Pulsatile	3	5	4	5	5	4	6	4	3	5	3	4
Model (4)												
Respiratory	4	5	3	5	5	5	1	6	4	5	5	7
Pulsatile	3	3	4	4	5	3	7	4	1	8	3	5
Total	12	15	12	20	25	15	7	24	4	40	15	35

Note that for model (4) with patients 7 and 9 the number of gates is one, since the amplitude of motion in both cases is negligible compared to one-half of scanner resolution.

**Figure 2:**
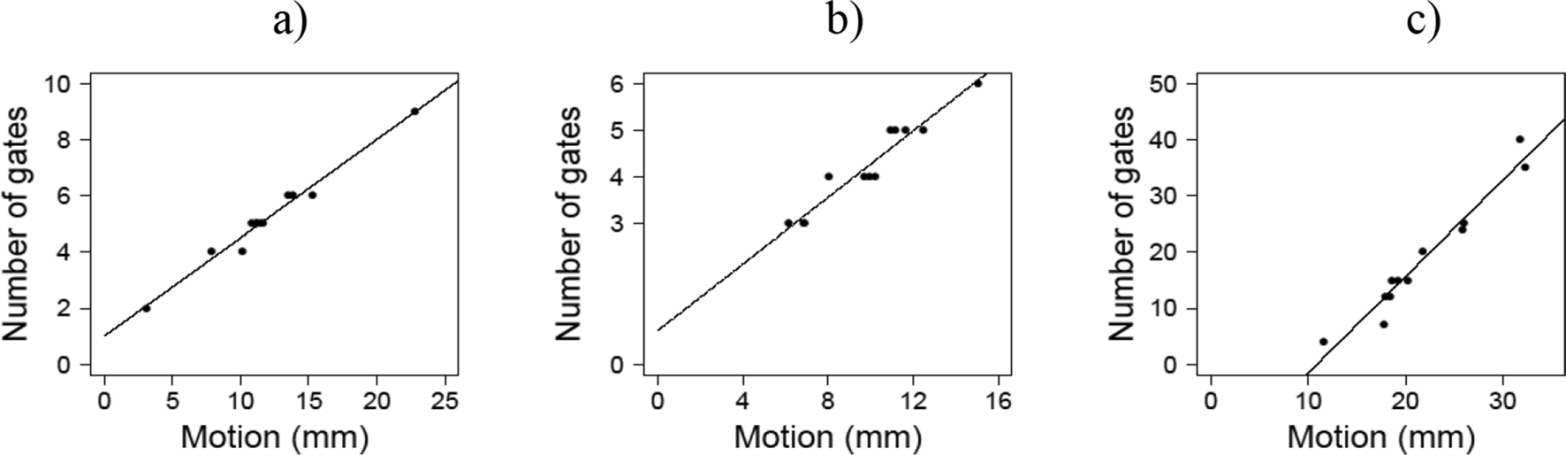
The optimal number of respiratory (**a**), cardiac (**b**) and dual (**c**) gates as a function of motion in mm (models (10), (11) and (12), respectively). Line is a linear fit of optimal number of gates (dots) as given in Table 6 and the motion given in Supplementary Data 2, describing the minimum number of gates needed for motion compensation in terms of the scanner resolution.

The results from the fitting to derive a general model for respiratory, cardiac and dual gates are shown in Fig. 2. Figure 2 represents the motion data of Supplementary Data 2 versus the modelled number of gates in Table 6 together with the regression lines presented in models (10), (11) and (12). As in^26^ we choose a linear function to be fitted for the data.

From the fitting, we obtained for optimal number of respiratory gates the following general model from models (1) and (3):

10 $${n}_{r}\left(m\right)=0.350*m+1.005,$$

where $m\ge 0$ is the amplitude of respiratory motion in mm. To derive the optimal number of cardiac gates we obtained the following general model from models (1) and (3):

11 $${n}_{p}\left(m\right)=0.356*m+0.718,$$

where $m\ge 0$ is the amplitude of cardiac motion in mm. Finally, for optimal number of dual gates using models (2) and (4) we obtained the following general model:

12 $${n}_{total}\left(m\right)=1.717*m-18.730,$$

where $m\ge 12$ is sum of the amplitudes of respiratory and cardiac motions in mm.

The correlation coefficient of the data and the fitted line for (10) is R^2^ = 0.978, for (11) is R^2^ = 0.926 and for (12) is R^2^ = 0.951. To determine the optimal number of dual gates, any of the models in (10), (11) and (12) can be used. Based on the R^2^ values the preferable method is to use (10) and (12).

Based on estimated motion in the patient study (Supplementary Data 2), the average respiratory motion was approximately 12 mm with cardiac motion of 10 mm. With these values, the optimal value of dual gates was solved from (12) and the number of respiratory gates from (10). The optimal number was 20 dual gates based on (12), corresponding optimally to 5 respiratory gates based on (10) and 4 cardiac gates based on the product ${r}_{opt}*{p}_{opt}$.

### Signal to noise ratio measurement

**Figure 3:**
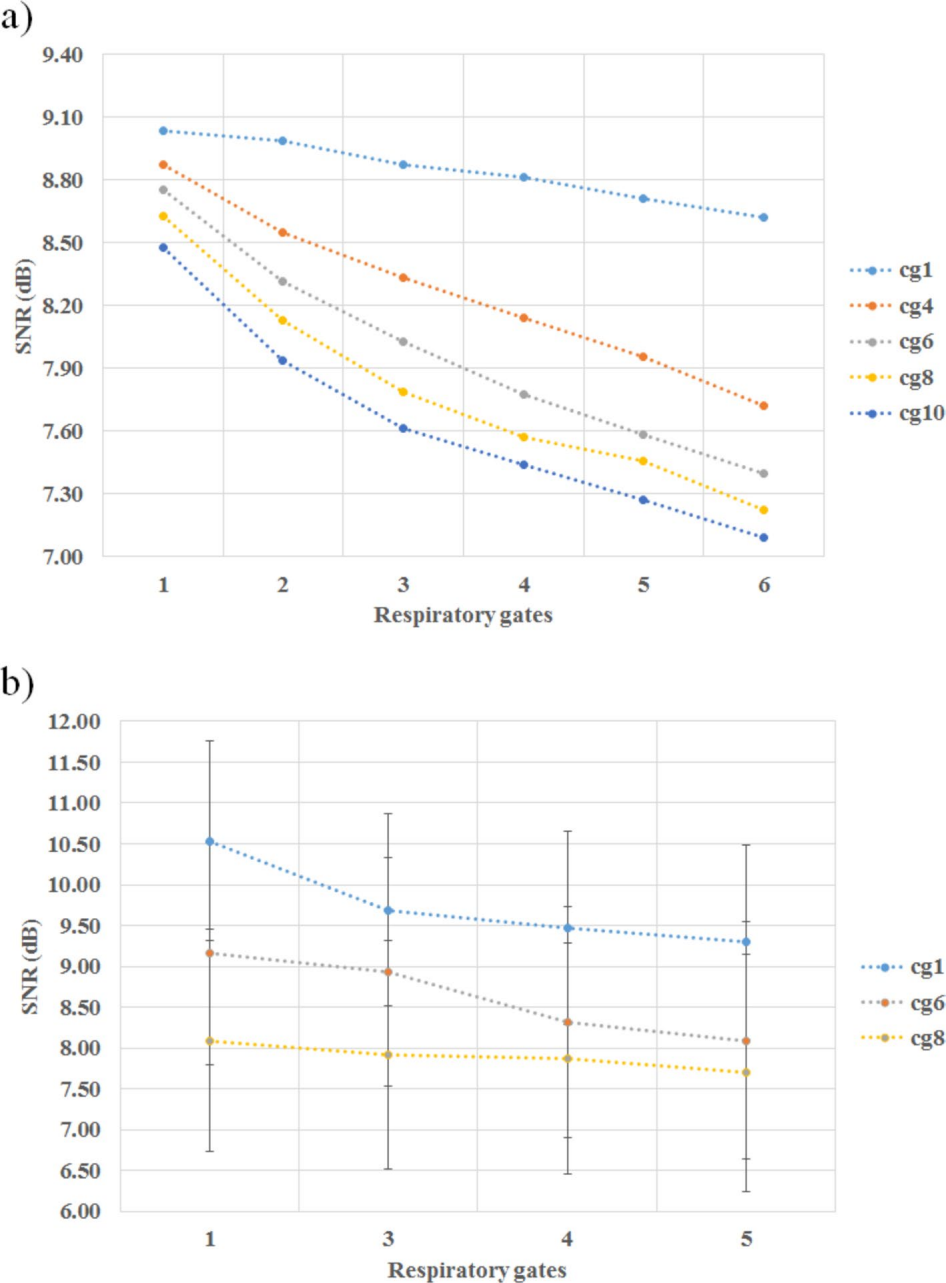
SNR for each gating scheme in the (**a**) phantom study and (**b**) patient study. The SNR is given as a function of respiratory gates where the individual line plots represent different cardiac gating schemes. The number of cardiac gates is 1, 4, 6, 8 and 10 for (**a**) and 1, 6 and 8 for (**b**).

**Figure 4:**
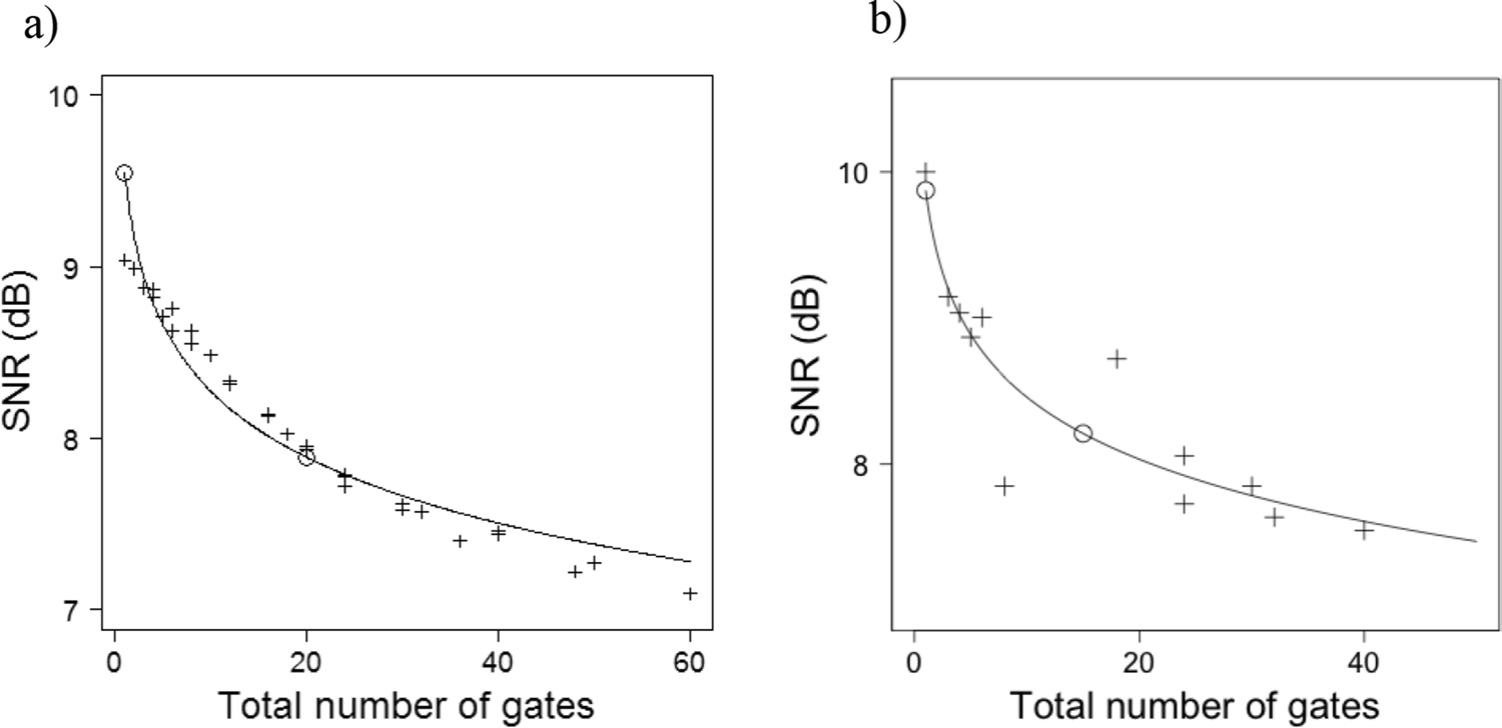
SNR versus the number of gates in the phantom study (**a**) and in the patient study for patient 11 (**b**). Plus signs indicate measured data, circles show SNR with no gating and optimal number of gates based on model (4) as in Table, and the curve describes the fitted SNR by equation $a+b*{\mathrm{log}}_{10}(n)$.

**Figure 5:**
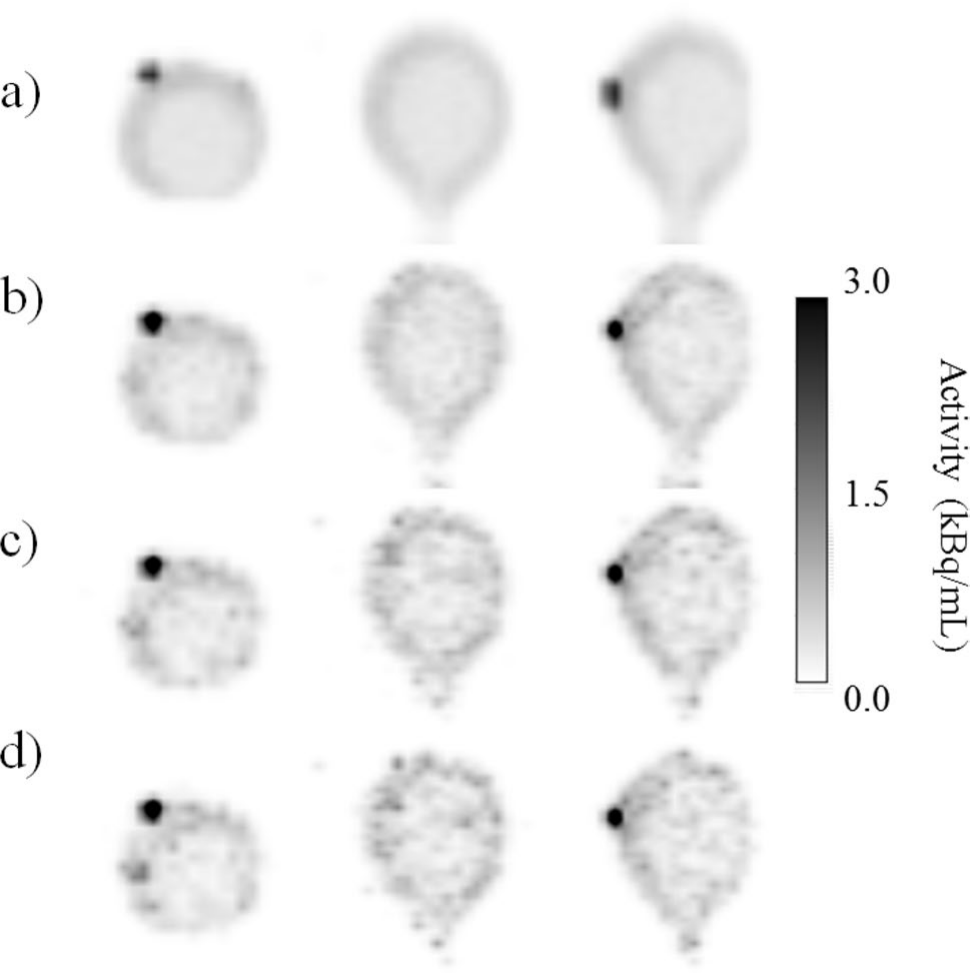
Comparison of different gating schemes with (**a**) non-gated and dual gated PET images with (**b**) three respiratory and three cardiac gates, (**c**) five respiratory and four pulsatile gates and (**d**) six respiratory and five pulsatile gates in the phantom study. The hot spot is blurred in the non-gated image versus dual gated images. The hot spot in the phantom is slightly smaller in (**c**) and (**d**) whereas (**d**) shows slightly increased noise compared to (**c**).

**Figure 6:**
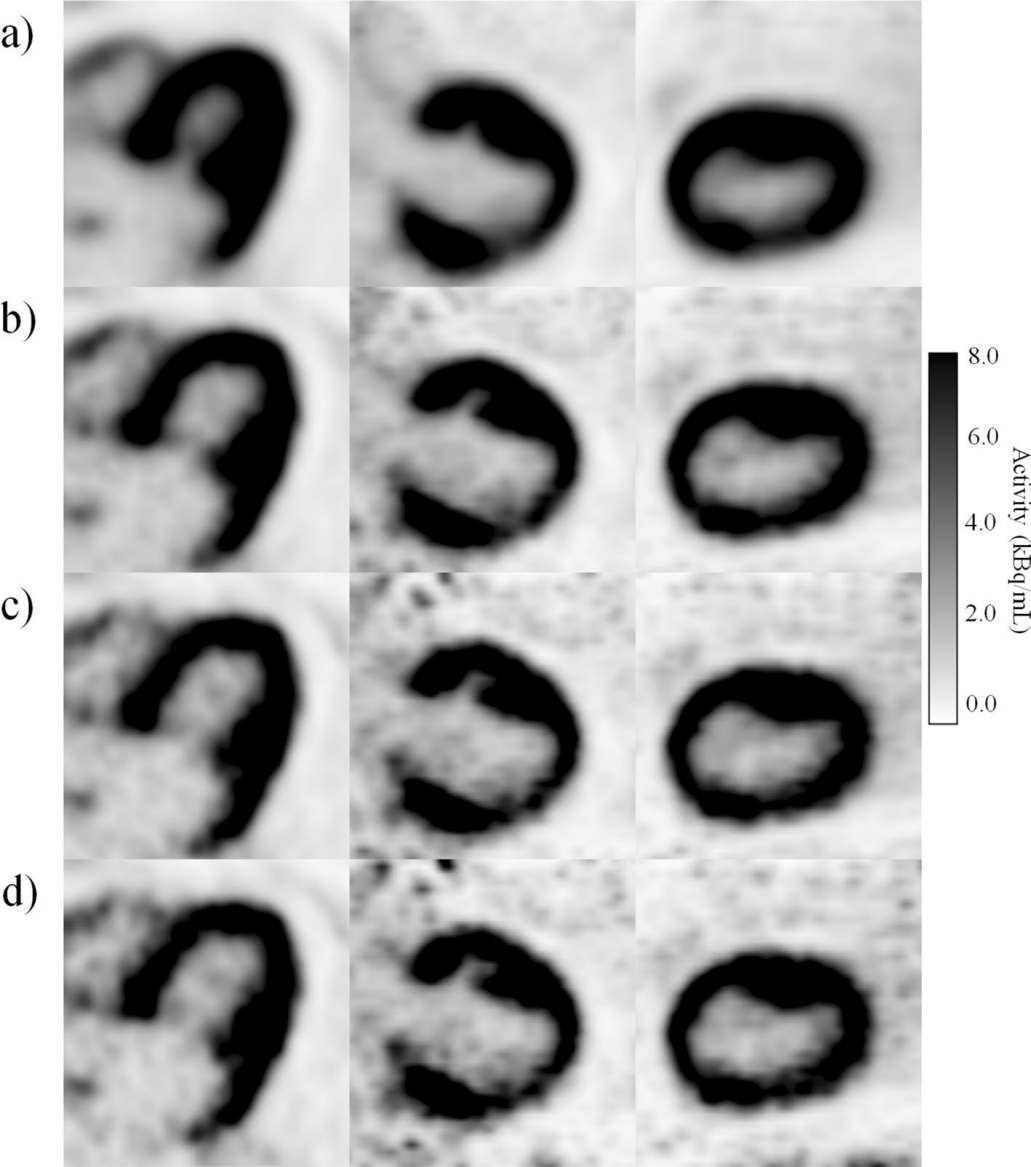
Comparison of different gating schemes with (**a**) non-gated and dual gated PET images with (**b**) three respiratory and three cardiac gates, (**c**) five respiratory and four pulsatile gates and (**d**) six respiratory and five pulsatile gates in the patient study, subject 11. The myocardium is slightly better delineated in (**c**) and (**d**). The difference between (**c**) and (**d**) is very small.

The SNR curves for the phantom and patient for different gating schemes are given in Fig. 3. The full table with all the SNR values for both the phantom and the subjects with all gating schemes is given in Supplementary Table 3. An example of fitted SNR curve for patient 11 is shown in Fig. 4. The RMSE between the fitted curve and the mean SNR is shown in Table 7. The mean RMSE of the fitted curve was 0.092 ± 0.043, which indicates that SNR can be evaluated accurately from the fit. Finally, examples of non-gated and dual gated images with the phantom and example patient (subject number 11) the optimal number of gates are shown in Figs. 5 and 6.

**Table 7:** The accuracy of the evaluated SNR fit measured with the RMSE (dB) of measured SNR and the SNR calculated from the fitted curve.

Patient number	1	3	4	5	6	8	9	10	11
RMSE (dB)	0.20	0.23	0.14	0.45	0.35	0.35	0.29	0.41	0.30

In the phantom study, the optimal number of gates was 5 respiratory and 4 cardiac gates. Difference between SNR of non-gated image and the dual gated image with the optimal number of gates was 1.7 dB in the phantom study.

In the patient study, the optimal number of gates was five respiratory and four cardiac gates as well. Using this optimal number of gates for motion minimisation in the patient study, SNR decreased by 2.1 ± 1.3 dB on average. Thus, using this number of gates fulfilled the criteria of SNR reduction with maximum of 3 dB.

### Evaluation of image quality

**Figure 7:**
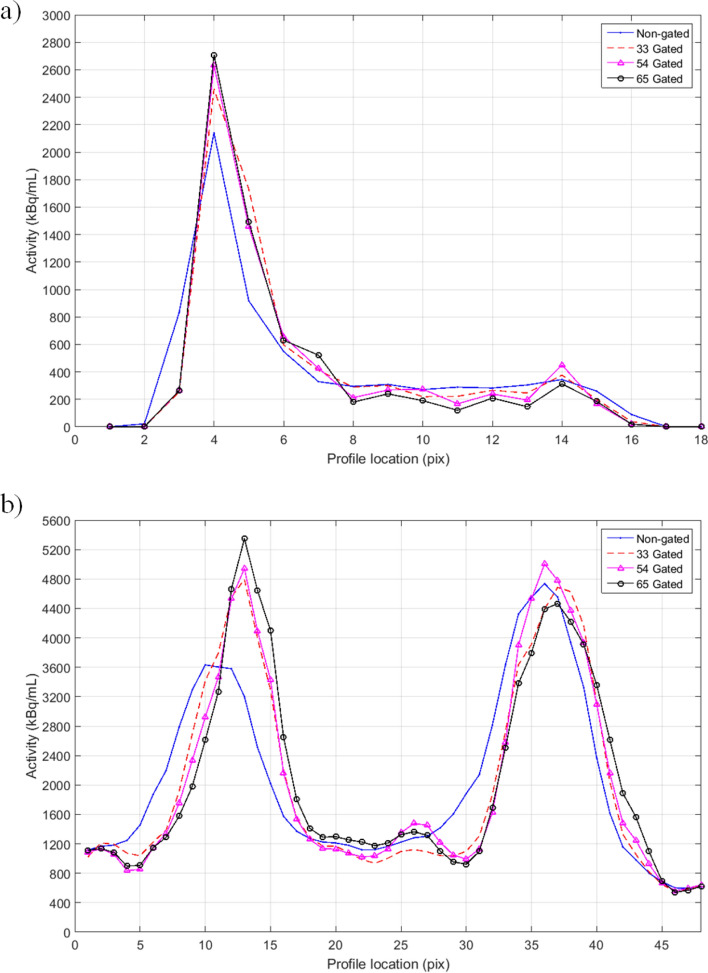
Example profiles of the (**a**) phantom over the hotspot in slice number 25 and (**b**) on subject 12 over the myocardium in slice number 23. The advantage of using five respiratory gates and four cardiac gates over three respiratory and three cardiac gates is better delineation of the hot spot profile and myocardium. However, increasing the number of gates over five respiratory and four cardiac gates does not result in significant increase in the profile peak.

The relative difference of FWHM in dual gated images versus non-gated images are presented in Table 8. Example profiles drawn to the most active hot spot in the phantom and in the myocardial wall of a single patient are depicted in Fig. 7.

**Table 8:** The relative differences (%) of FWHM in the patient study between dual gated (5 respiratory and 4 cardiac gates) and non-gated images.

Patient	1	2	3	4	5	6	7	8	9	10	11	12
Septal FWHM (mm)	− 9.2	− 19.6	− 12.7	− 8.3	− 5.9	− 9.0	− 13.6	− 13.1	− 58.8	− 19.3	− 10.4	− 39.2
Lateral FWHM (mm)	− 18.0	− 53.4	− 10.3	− 38.1	− 25.3	− 10.0	− 11.8	− 38.2	− 42.3	− 15.7	− 21.2	− 10.7

Reduction of the FWHM profiles is shown for both the septal and lateral wall of the myocardium.

In the phantom study, the diameter of the most active hot spot was reduced by 24% in the dual gated images compared to non-gated images. In the patient study, the thickness of myocardium wall was reduced on average by 21%. Average value the FWHM value of the thickness of myocardium wall in non-gated images was 35 mm and 27 mm in dual gated images.

### Comparison with an existing motion model

Table 9 shows the model fitting results from model (9) in the phantom study in comparison to model (1). Models (1) and (9) have very similar performance in terms of estimated motion in the phantom study.

**Table 9:** Error in mm of respiratory motion given by models (1) and (9) compared to the reference motion from the phantom.

Model	Motion	Target	RMSE	MD	Absolute value
(1)	Respiratory	Largest hot spot	0.38	0.30	0.00–0.73
(1)	Respiratory	Average of all	0.44	0.35	0.00–0.78
(9)	Respiratory	Largest hot spot	0.33	0.28	0.02–0.73
(9)	Respiratory	Average of all	0.38	0.35	0.05–0.69

Absolute value refers to the absolute difference of estimated motion from the model and the reference motion.

Using (1) and (9), a general model can be derived where the number of gates can be obtained as function of estimated respiratory motion. Based on (1), (9) and their data, Dawood et al*.* reported the following linear fit for the optimal number of respiratory gates, expressed below with a similar convention as models (10) to (12):

13 $${n}_{1}\left(m\right)=0.488*m-0.869,$$

where ${n}_{1}$ is the number of gates and *m* is the respiratory motion in millimetres. For our data the corresponding linear fit is:

14 $${n}_{2}\left(m\right)=0.407*m-0.408.$$

**Figure 8:**
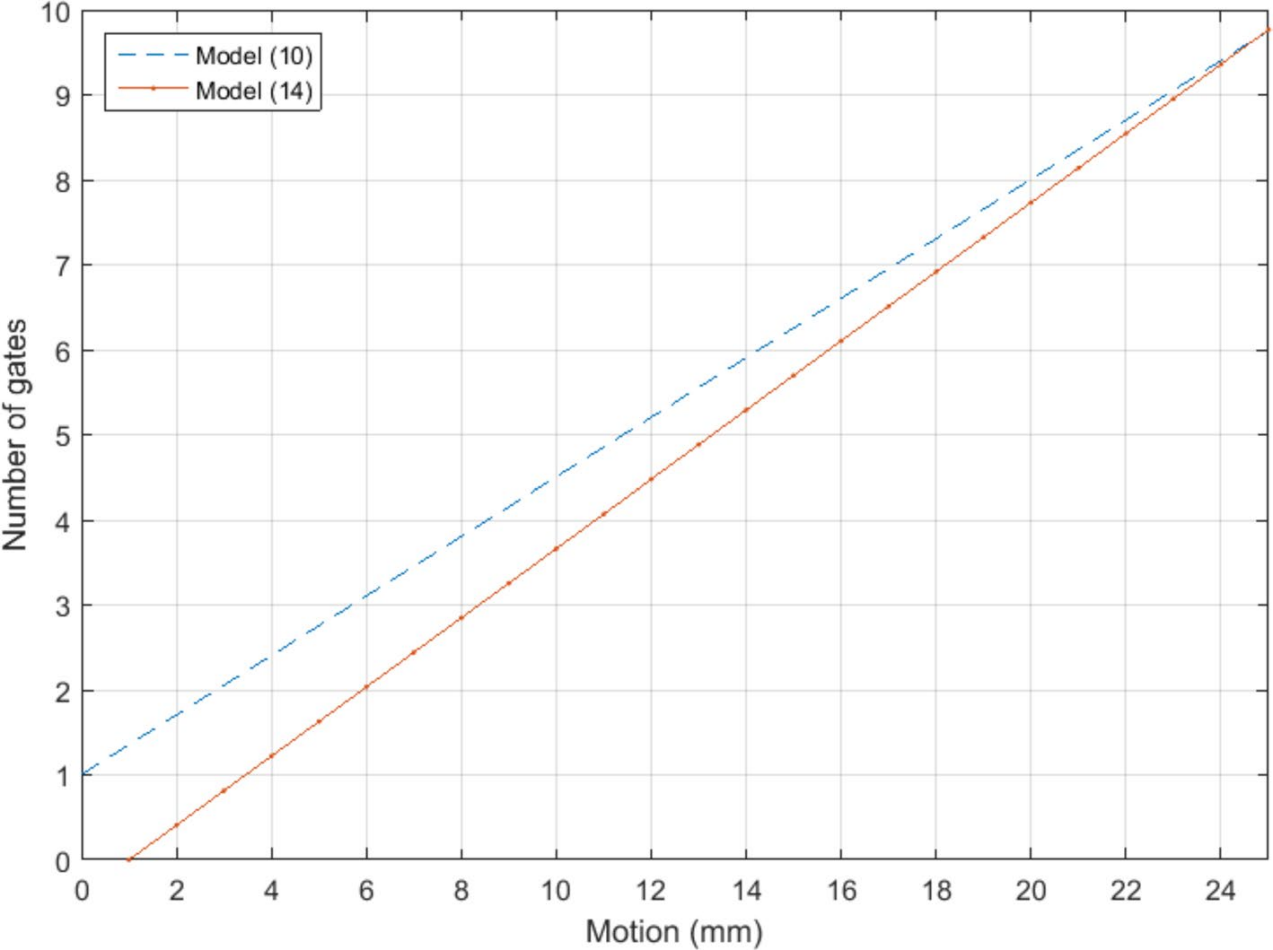
Linear functions for the optimal number of gates as a function of the amount of motion. Red solid line is fit for Dawood’s model as in (14), and blue dashed line is for our model as in (10). Both functions show similar behaviour over the motion range in this study.

The behaviour of the linear functions for the model in (14) and our proposed model for respiratory gating (10) are illustrated in Fig. 8.

## Discussion

In this study, two motion models for motion estimation were presented, validated and applied to respiratory, cardiac and dual gating in PET/CT imaging of the vulnerable plaque. Models were built using phantom and patient studies and the motion estimation accuracy was determined against the known motion in the phantom and the motion measured from patient images. The models were then used to define optimal number of dual gates. The effect of the optimal number of gates to image quality was quantified using automated SNR computation and profiles drawn in manually selected regions. While research of dual gated cardiac PET imaging continues to be active, this study is the first attempt to define optimal number of dual gates using two new motion models. To the best knowledge to the authors, no similar attempt has been made previously to derive a dual respiratory-cardiac motion model.

### Discussion of the results

We introduced a model for respiratory and cardiac motion (1), and a model for dual motion (2). In terms of motion estimation performance, the generalised model (2) for dual gating gave very similar results to the model (1) both in respiratory and cardiac motion. The differences in estimated motion versus true motion with both models was minimal in the phantom study (Table 3) and in the patient study (Table 4). Model (1) has slightly higher accuracy in motion estimation compared to model (2) in the phantom study, however the maximum difference between the models is below 2 mm.

Based on estimated motion in the patient study (Supplementary Data 2), the respiratory motion on average was less than 12 mm with cardiac motion less than 10 mm. Altogether, our estimates are in agreement with previous reports and with our phantom study^19,25^. For example, Daou^1^ reported respiratory motion of 10.5 mm with 41 patients, while Dawood et al. (2009) reported motion of 11.5 mm in nine patients. Finally, coronary motions of 8 to 23 mm on have been reported in MRI imaging^39^. It can be seen that patients 8, 9 and 10 with low myocardium activity, the motion estimation accuracy is reduced in model (2). However, at maximum this difference is less than 3.5 mm, which is smaller than the resolution of the PET system used in this study.

In terms of estimating the optimal number of gates in the phantom study, the measured motion increases more efficiently by increasing number of respiratory gates than cardiac gates (Table 4). This is due to a simple reason that the respiratory motion amplitude is much larger (20 mm vs. 7 mm) compared to the cardiac motion in the phantom. An intercept point for optimal motion minimisation was defined at five respiratory and four cardiac gates, after which adding more gates does not increase the estimated total motion (Table 4). Although it might seem, that the number of gates could be interchanged to produce the nearly same amount of motion, they should be optimised in terms of individual cardiac and respiratory motion amplitudes.

In terms of estimation of the optimal number of gates in the patient study, both the model (3) and model (4) gave very similar results, with maximum difference of one gate (Table 6). Whereas the differences between the models are small, there is more variation on the number of gates patient-to-patient basis. This is expected, since the motion amplitudes in both respiratory and cardiac motions between patients varied from 2.5 mm to 17.8 mm and 5.5 mm to 13.0 mm due to physiological effects. In two cases (subjects 7 and 9), model (4) resulted in optimal number of gates as 1, with 1 respiratory gate for subject 7 and 1 pulsatile gate for subject 9. In these two cases, the motions were 2.5 mm for respiratory motion in subject 7 and 5.5 mm for pulsatile motion in subject 9. As both motions are below or close to the 2.6 mm threshold which is defined by the criteria (${m}_{est}-s/2$) used to derive Eqs. (3), (4) the models assume that there is no need to minimize the motion, producing an optimal gate number of 1.

The largest difference in the number of gates was seen with patient 10, where using model (4) resulted in a larger number of total gates. As the estimated motion was also largest with this patient with model (2), it seems that errors in motion estimation might propagate to model (4) when estimating the number of gates. However, it should be noted that while in model (3) the number of respiratory and cardiac gates needs to be determined separately, model (4) gives the total number of dual gates directly. Whereas these models could be used directly to optimise the number of gates on patient-to-patient basis for individual motion amplitudes, the optimal number of gates versus motion needs to be generalised for further application. This was determined based on deriving general models (10) to (12) from Fig. 2.

In deriving a general model to be applicable on this patient group and to other patient studies outside this group, we used motion models (1) and (2) to define estimated motion and the optimal number of gates from (3) and (4) in the patient study for each subject. This information was then used to build general models (10), (11) and (12) for number of gates as function of motion amplitude using linear regression. The results of Section “Determining the optimal number of gates as function of motion—patient study” suggest that the preferred way of defining the optimal number of dual gates is to use (10) to define the optimal number of respiratory gates, and (12) to define the optimal number of dual gates. Quotient of these gives the optimal number of cardiac gates. For simplicity, (12) can be also applied directly. By assuming 12 mm and 10 mm for respiratory and cardiac motions, the optimal number of gates was determined as 5 respiratory and 4 cardiac gates which is in accordance with the number of gates (5 and 4) derived in the phantom study (Table 4). This number of gates can be applied to minimise respiratory motion up to 12 mm and cardiac motion of 10 mm across various patient groups outside of this study.

The image quality was assessed by SNR calculations, visual analysis and measurement of FWHM. A dual gated image with optimal number of dual gates was compared with the non-gated image. The SNR analysis clearly shows that increasing the number of gates further from the optimal will not be beneficial for additional motion capture and will only result in decreased SNR (Figs. 3 and 4). This SNR decrease can be visually determined from the phantom and patient images (Fig. s5 and 6). The SNR could be also fitted over the individual patients accurately as in the previous study^26^. There are small variations in the accuracy of the fit over individual patients, as shown in Table 7. It can be seen that patients with high body weight (> 90 kg) tend to show lower SNR compared to others. Thus, the variation in SNR in these patients is expected as it is generally observed that patients with high body weight have reduced SNR in PET imaging.

The optimal dual gating resulted in improved definition of the hot target in the phantom images and clearer definition of the myocardium in the patient images (Fig. 7). In both phantom and patient study, the image quality in terms of FWHM improved more than 20%, while the SNR decreased about 20%, when comparison was done in dB. This SNR loss could be accounted for by implementing a motion correction method to sum up the dual gates or to implement non-equal time bins in both respiratory and cardiac gating.

In comparison to a previously introduced respiratory-only model, the proposed model (1) had equal performance (Fig. 8). The motion estimates of the models versus the reference motion in the phantom are very close to each other, with differences smaller than 0.10 mm (Table 9). There is a difference of approximately 1 gate when the motion is below 20 mm, which is explained by the assumption that for motion of 0 mm, the number of gates should also equal 1 for model (1). Compared to the model introduced in Dawood et al*.*, the model (1) is also mathematically simpler and can also be applied to cardiac motion. As there are no respiratory-cardiac motion models introduced previously, we could perform the comparison with respiratory motion modelling only.

### Generalising the model to new datasets and new PET/CT systems

First, our intent was to define the optimal number of gates which could be applied directly in clinical practice without further motion modelling or gating. Thus, applying the gates defined in this study should be applicable to various patient groups, given the fact that in most cases 5 respiratory gates and 4 cardiac gates are sufficient to minimise respiratory motion up to 12 mm and cardiac motion up 10 mm, corresponding to total motion of 22 mm. This amount should be enough to capture the motion accurately in most routine patients. In cases of unusually large motion this number might be increased as required. As the motion amplitude also varies between patients depending on the breathing and cardiac pattern, it is always better to fix the number of gates across a patient group in a way that it offers the optimal motion minimisation in majority of cases in regard to the loss in image quality.

In applying the models to a new patient group, one needs only to measure the motion within the new dataset (either patient-wise or an average of the group) and apply the motion *m* to Eqs. (10) to (12). There is no need to perform reformulation of the model. Also, in applying the models to the new data there is no need to perform all the gating combinations used in this paper, only to perform gating once with high number of gates, e.g. 10 respiratory and cardiac gates, and then apply the measured motion in models (10) to (12). However, new reconstructions are needed with motion measurements unless the motion can be recovered by other means.

Without image reconstruction, the amount of motion could be measured with a quantifiable respiratory signal, which has been determined to correspond to a particular amount of motion. For example, our group has previously shown^30^ that you could potentially use the spirometry signal in this study to derive a motion estimate based on the signal alone, as there is a linear dependence between the volume change in the spirometry signal and CMA motion. This would allow to determine the optimal number of gates based on the respiratory signal alone, using a simple linear equation to estimate the motion *m*, according to Kokki et al*.*^30^.

The proposed models in Eqs. (1) to (4) are also very simple, with only two parameters as input: the total motion measured from images and the number of gates used for gating. This makes applying them to new datasets straightforward and robust, as the only source of uncertainty comes from the accuracy of the motion measurement. However, a PET/CT system-dependent parameter in the model is the system resolution, meaning that for very high resolution PET/CT systems the number of gates would be needed to be increased. The model should not be sensitive to other system- or reconstruction specific qualities, such as matrix size, filtering, time-of-flight (TOF) imaging or point spread function (PSF), unless they alter the amount of estimated total motion significantly.

In terms of model accuracy, any error in the data which affects the measurement of the total motion in the images will have an effect to the number of gates derived with the model. Regular breathing irregularities will tend to be averaged within the gated images and should not have a large effect to the model accuracy. Since the total motion is also measured between the motion extremes, the model is not very sensitive to small changes in the respiratory or cardiac cycles.

However, a large gross motion of the patient might change the total motion and currently the model does not account for that. This might result in over- or underestimation of the number of gates with the patients with large gross motion depending on the direction and magnitude of the repositioning event. With patients 2, 6 and 7 gross motion on the RPM signal was seen. However, on patients 2 and 6 there were no ill effects on the estimated number of gates. On patient 7, the atypical accumulation and a large fraction of lost signal (> 40%) were determined to be the main factors affecting motion estimation as opposed to gross motion.

### Limitations

Our study has a limitation which concern the application of different gating methods. The current study did not compare the effect different gating methods to the preserved amount of data in each gate. For example, in cardiac gating, phase-based cardiac gating is the clinical standard, but with different gate durations. Kokki et al*.*^19^ used four cardiac gates and the longest gate contained half of the data. As there are numerous ways in determining the division of both respiratory and cardiac gates, it would be of interest to study the optimal division of the gates. Thus, by dividing the amount of data differently, more data could be preserved in the most optimal gate. However, it should be noted that this only concerns the division of gate lengths, not the model itself. Any gating scheme will allow to derive the model as long as motion between two motion extremes in respiratory or cardiac states can be measured and given as input to the model.

Second, the ground truth motion estimates from the patient studies could only be derived based on patient PET images. The motion was determined by using the motion of CMA measured from PET images^30^. Ideally, it would be beneficial to estimate the number of gates also from external signals^30^, although this method has limitations as well, discussed in^40^. While CMA is limited in accuracy by resolution of the PET images, it so far offers a viable solution for image-based motion determination for defining the optimal number of gates when number of images to analyse is large (over 1000 in this study) and has been used previously in several studies^22,40^. As we used respiratory- or cardiac-averaged images to derive motion estimates, small differences in the uptake are averaged out for accurate motion measurements. The method is limited as it only detects displacement, not rotation or deformation and depending on the segmentation quality, only a part of the heart might be detected. However, since we measure motion between the two motion extremes, this method is also in a sense, robust to changes in also atypical uptake in the myocardium. Potentially, MRI might be used in a future study as an alternative, non-invasive option for as a ground truth measurement for motion^41^. However, none of the patients in this study cohort undergone MRI.

In addition, this study was conducted on the Discovery VCT system which does not incorporate TOF imaging or PSF modelling. Addition of TOF and PSF would result in improved image quality and hence, a higher number of dual gates might be used to achieve the same image quality. On the other hand, the same number of gates could be directly used with PSF and TOF to achieve the same motion minimisation but improved image quality in each gate. According to^42^, TOF improves the SNR in the images, whereas using PSF would give also a better definition of the small targets in the image^43^. Using the number of gates specified in this paper with a more recent PET/CT system would result in improved image quality if TOF and PSF would be included. Thus, further studies for assessing recent reconstruction methods for optimal gating are encouraged.

Finally, the presented motion models and the methodology described in this paper offer simple, easy-to-apply alternatives to derive motion estimates, which could be used to optimise both motion minimisation, the number of gates and image quality across different PET systems. To apply the results in this study from a new dataset, one needs only to measure the motion within the dataset (either patient-wise or an average of the group) and apply the measured motion *m* to Eqs. (10) to (12). We believe that the methodology presented in this paper with the general models offer a solution for determining the optimal number of gates which are straightforward to apply in practice and are low in computational complexity.

Further assessment of the methodology described in this paper should be performed with larger patient population in regard to optimal coronary plaque detection. To adopt the study materials in practice, patients with larger motion amplitude should also be included. Thus, further studies are encouraged on potential application of the simple models to motion tracking, optimisation of the number of gates and motion correction in PET imaging.

## Conclusions

We introduced and validated two motion models which can be used for accurately estimating respiratory and cardiac motion in dual gating using spirometry data. The motion estimates from the models can be applied for determining the optimal number of gates for respiratory, cardiac and dual gating. Based on the models, the optimal number of dual gates for minimising both respiratory and cardiac motion effects was derived, consisting of five respiratory and four cardiac gates. This number of gates can be applied to minimise respiratory motion up to 12 mm and cardiac motion of 10 mm, corresponding to total motion of 22 mm across various patient groups.

## Supplementary information

Supplementary Information 1.

Supplementary Information 2.

Supplementary Information 3.

Supplementary Information 4.

## Data Availability

The datasets generated during and/or analysed during the current study are available from the corresponding author on reasonable request.
