# Supplementary material for: Estimation of optimal number of gates in dual gated 18F-FDG cardiac PET
Source: Sci Rep. 2020 Nov 9;10:19362. doi: 10.1038/s41598-020-75613-5 (PMC7653943; doi:10.1038/s41598-020-75613-5)
Supplement: Supplementary file 1 — Supplementary Information 1. [file 41598_2020_75613_MOESM1_ESM.docx]

# Estimation of Optimal Number of Gates in Dual Gated ^18^F-FDG Cardiac PET

R. Klén^1^, J. Teuho^1^, T. Noponen^1,2^, K. Thielemans^4,5^, E. Hoppela^1^, E. Lehtonen^6^, H.T. Sipila^1^, M. Teräs^3,7^, J. Knuuti^1^

^1^Turku PET Centre, University of Turku and Turku University Hospital, Turku, Finland

^2^Department of Clinical Physiology and Nuclear Medicine, Turku University Hospital, Turku, Finland

^3^Department of Medical Physics, Division of Medical Imaging, Turku University Hospital, Turku, Finland

^4^University College London, London, United Kingdom

^5^Hammersmith Imanet Ltd, London, United Kingdom

^6^Department of Future Technologies, University of Turku, Turku, Finland

^7^Institute of Biomedicine, University of Turku, Turku, Finland

These authors contributed equally: R. Klén and J. Teuho.

Supplementary data

**Applying the analytical model to estimate the optimal number of gates**

An example of model (1) and the value $m_{est}$ are depicted as a function of gates in Fig. S1. After automated image segmentation and measurement of motion with different number of gates, the motion as a function of gates can be fitted using either models (1) or (2) as shown in Fig. S1 (black curve). Thereafter, model (1) can be used to estimate total motion as asymptotic value $m_{est}=\lim_{r\to\infty} f(r)$. Because value $m_{est}$ is asymptotic, we define our target motion to be $m_{est}-s/2$, where $s$ is the scanner resolution. The value $m_{est}-s/2$ is illustrated in Fig. S1 (grey line).


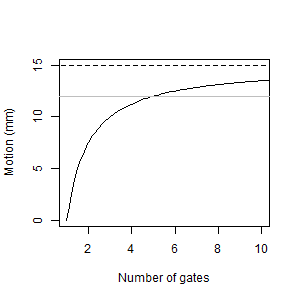


**Figure S1.** Example of behaviour of model (1) and optimal number of gates. Black curve is the motion given by model (1), black dashed line is the total amplitude of the motion based on the model ($m_{est}$), and grey line is the motion used for optimal number of gates ($m_{est}-s/2$).

**Supplementary Data 1.** Motion measured in millimetres from gated PET images in the phantom study.

**Supplementary Data 2.** Motion measured in millimetres from gated PET images in the patient study.

**Supplementary Data 3.** Measured SNR with the phantom and patient studies of all the gating schemes with the fitting coefficients for the SNR curve.
